# Novel Imaging and Genetic Risk Markers in Takotsubo Syndrome

**DOI:** 10.3389/fcvm.2021.703418

**Published:** 2021-08-17

**Authors:** Luca Arcari, Luca Rosario Limite, Carmen Adduci, Matteo Sclafani, Giacomo Tini, Francesca Palano, Pietro Cosentino, Ernesto Cristiano, Luca Cacciotti, Domitilla Russo, Speranza Rubattu, Massimo Volpe, Camillo Autore, Maria Beatrice Musumeci, Pietro Francia

**Affiliations:** ^1^Cardiology Unit, Mother Giuseppina Vannini Hospital, Rome, Italy; ^2^Department of Cardiac Electrophysiology and Arrhythmology, IRCCS San Raffaele Scientific Institute, Milan, Italy; ^3^Cardiology, Clinical and Molecular Medicine Department, Faculty of Medicine and Psychology, Sapienza University of Rome, Rome, Italy; ^4^IRCCS Neuromed, Pozzilli, Italy

**Keywords:** takotsubo, cardiac magnetic resonance imaging, T1 mapping, T2 mapping, speckle tracking echocardiography, particle imaging velocimetry, genetic, prognosis

## Abstract

Takotsubo syndrome (TTS) is an increasingly recognized condition burdened by significant acute and long-term adverse events. The availability of novel techniques expanded the knowledge on TTS and allowed a more accurate risk-stratification, potentially guiding clinical management. The present review aims to summarize the recent advances in TTS prognostic evaluation with a specific focus on novel imaging and genetic markers. Parametric deformation analysis by speckle-tracking echocardiography, as well as tissue characterization by cardiac magnetic resonance imaging T1 and T2 mapping techniques, currently appear the most clinically valuable applications. Notwithstanding, computed tomography and nuclear imaging studies provided limited but promising data. A genetic predisposition to TTS has been hypothesized, though available evidence is still not sufficient. Although a genetic predisposition appears likely, further studies are needed to fully characterize the genetic background of TTS, in order to identify genetic markers that could assist in predicting disease recurrences and help in familial screening.

## Introduction

Takotsubo syndrome (TTS) is an acute heart failure (HF) syndrome accounting for ~1-2% of all suspected acute coronary syndromes, it is characterized by transient systolic dysfunction (1) and burdened by a significant rate of in-hospital and long-term mortality (2). Several diagnostic criteria have been proposed, all of which included the presence of transient left and/or right ventricular wall motion abnormalities, ECG changes and increased cardiac biomarkers with the absence of any identifiable culprit coronary artery disease (CAD) (1, 3, 4). In recent years, the clinical knowledge of TTS has progressively improved owing to growing awareness, increased reported prevalence (5), and the availability of a number of novel diagnostic tools and prognostic markers. The present review aims to summarize the recent advances in TTS risk stratification with a specific focus on novel imaging and genetic markers.

## Outcome of Takotsubo Syndrome

TTS was initially perceived as a benign condition (6, 7). This was likely due to the limited sample size of early population studies, which mostly included “classic” TTS patients and presentations (e.g., post-menopausal women experiencing an emotional stress). This subset of patients was indeed later recognized as being at lower risk of adverse outcomes (8, 9).

To date, multiple studies from several multicenter registries have led to a changing perspective and currently agree on considering TTS a not-entirely benign condition, burdened by both in-hospital and long-term mortality (2, 9–12). Several acute in-hospital complications of TTS have also been described, including acute HF, arrhythmias, and thromboembolic events (13, 14) (Table 1). The reported incidence of HF syndromes (including severe pulmonary edema and/or cardiogenic shock) ranges from 6 to 20% (15, 16). In these cases, TTS may even require inotropic or mechanical circulatory support (1). Also, life-threatening arrhythmias (complete atrioventricular block, ventricular arrhythmias, and cardiac arrest) may occur during the acute phase of TTS in up to 8% of the cases (17). Left ventricular (LV) thrombi secondary to apical akinesia have been reported in 2.5% of TTS patients and are associated with cardioembolic complications such as stroke or transient ischemic attacks (18).

**Table 1:** A table summarizing the major acute complications of patients with TTS.

**Acute complication**	**Frequency**
Acute heart failure	12-45%
RV involvement	18-34%
LV outflow tract obstruction	10-25%
Mitral regurgitation	14-25%
Cardiogenic shock	6-20%
LV thrombus	2-8%
Ventricular arrhythmias	4-9%
In-hospital death	1-5%
Cardiac rupture	<1%

*Rates taken from (1). RV, right ventricle; LV, left ventricular*.

Despite TTS is characterized by a substantial recovery of LV systolic function, studies with long-term follow-up have unraveled adverse outcomes, with rates of morbidity and mortality comparable to those experienced after an acute myocardial infarction (AMI). Stiermaier et al. reported a long-term mortality higher than in patients with ST-elevation AMI (19). In the SWEDEHEART registry, mid-term (median 25 months) mortality after TTS was similar to that of AMI (adjusted hazard ratio: 1.01, 95% CI 0.70–1.46, *p* = 0.955) (20). In the InterTAK registry, long-term rate of death from any cause was 5.6% per patient-year and that of major adverse cardiac and cerebrovascular events was 9.9% per patient-year, similar to that observed in a matched control-group of AMI patients (10). Likewise, the Italian TIN registry reported that TTS long-term mortality was comparable to a propensity score-matched cohort of AMI patients (21). Concordant data with comparable TTS mortality rates were reported by the GEIST (22) and RETAKO (23) registries.

Finally, the risk of TTS recurrence is low but persistent throughout the clinical history of TTS. This is a potentially life-threatening event, presenting even several years after the first occurrence (24). In this view, TTS should not be regarded as a benign disease, neither in the short- nor in the long-term (25). Accordingly, dedicated follow-up after the index event is strongly suggested (1).

Over the years, several studies have investigated the usefulness of diverse tools (particularly imaging) to better define risk stratification in TTS (Table 2).

**Table 2:** A summary comparing the main diagnostic features and risk markers (established or emerging) of different imaging modalities.

**Imaging modality**	**Diagnostic features**	**Established risk markers**	**Emerging risk markers**
**Echocardiography**	- Transient circumferential wall motion abnormalities (except focal type)	- LV ejection fraction - RV involvement - LV outflow tract obstruction - Mitral regurgitation	- Atria and ventricles deformation analysis by speckle tracking
**CMR**	- Transient circumferential wall motion abnormalities (except focal type) - Increased native T1, T2 and ECV - Absence of LGE	- LV ejection fraction - RV involvement	- T1 and T2 mapping - Atria and ventricles deformation analysis by feature tracking - Brain activity by functional MR imaging
**CT**	- Absence of culprit CAD	- CAD	- pFAI
**Nuclear imaging**	–	–	- Reduced 123-I-MIBG uptake - Brain activation, amygdala activity (TTS recurrence)

*CMR, cardiac magnetic resonance; CT, computed tomography; ECV, extra-cellular volume; LV, left ventricular; RV, right ventricle; CAD, coronary artery disease; pFAI, peri-coronary fat attenuation index*.

### Imaging Markers

#### Echocardiography

In the acute phase of TTS, transthoracic echocardiography (TTE) is the cornerstone of risk stratification. Several features can be identified by standard TTE that are associated with a higher risk, including low LV ejection fraction (EF) (26, 27) and right ventricular (RV) involvement (2). Echocardiography might be helpful in identifying the ballooning pattern, although the negative prognostic value of apical ballooning as compared to atypical forms is still debated (28–31). Furthermore, TTE allows to identify acute complications of TTS that have been related to prognosis, such as LV outflow tract obstruction and functional mitral regurgitation (11, 32, 33).

Novel, advanced echocardiographic techniques may provide an accurate and sensitive detection of cardiac abnormalities during both the acute and the “recovery” phase of TTS. Speckle tracking-TTE (ST-TTE) provides parametric quantification of myocardial chambers deformation. In the acute phase of TTS, assessment of LV global longitudinal strain (GLS) by ST-TTE has an important prognostic value, being associated to worse outcomes including in-hospital death and major adverse cardiovascular events (34). Also RV assessment by ST-TTE can be useful, as RV-GLS outperform tricuspid annular plane systolic excursion in identifying RV dysfunction (35). Finally, left atrial deformation has been described as transiently impaired in the acute phase of TTS, suggesting a cardiac involvement extending beyond the visually assessed areas of abnormal ventricular wall motion, and its finding has been associated with a higher rate of in-hospital complications (36).

**Figure 1:**
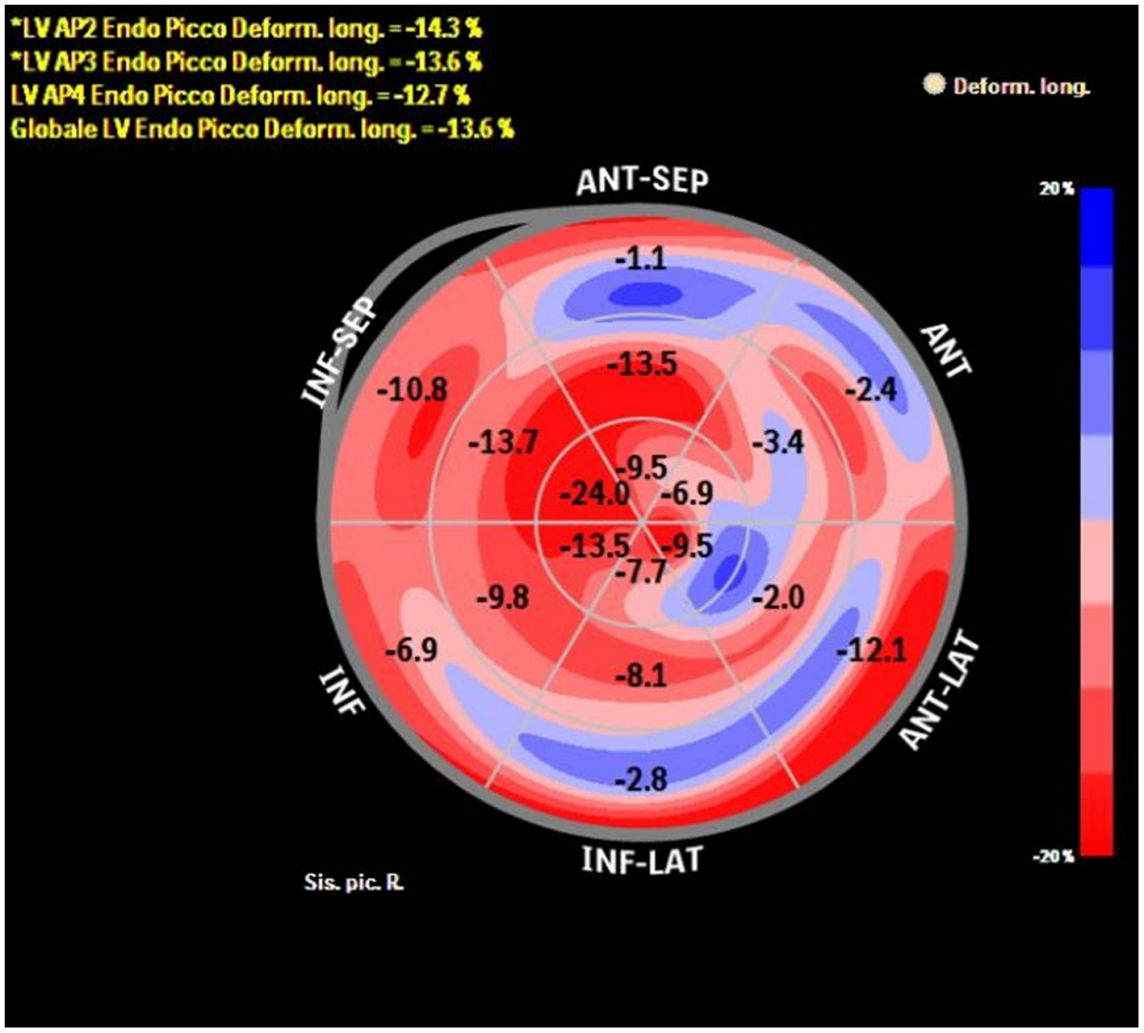
Bull-eye depicting longitudinal strain of the LV by speckle tracking echocardiography in a patient with previous TTS at 1-month follow-up after the acute event. Despite complete recovery of LVEF, longitudinal systolic function is still impaired (global longitudinal strain −13.6%).

The added value of ST-TTE is even more evident after the acute event, as LVEF usually normalize, but functional LV abnormalities might persist (Figure 1). Indeed, within the first few months after TTS hospitalization, LV-GLS is impaired despite normalization of LVEF (37, 38), and a reduced LV-GLS at 3 months after TTS associated with both persistent elevation of natriuretic peptides and impaired physical exercise capacity (39). Furthermore, by means of ST-TTE, impaired LV systolic longitudinal function has been observed over 1 year after the acute event, questioning the common perception of TTS as a transient condition (40). However, larger long-term data of the prognostic usefulness of ST-TTE in TTS are currently lacking.

**Figure 2:**
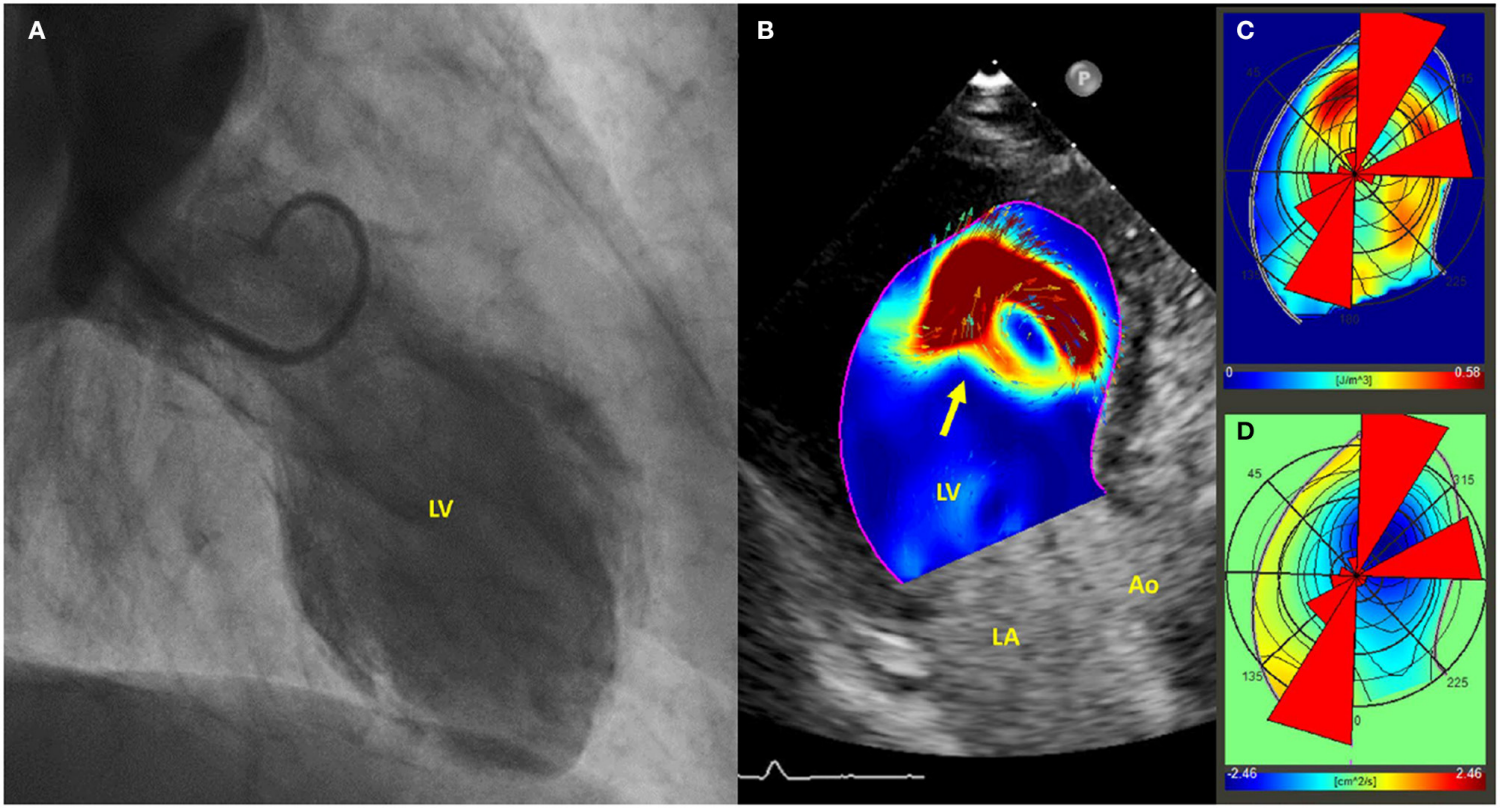
**(A)** Left ventriculogram demonstrating apical ballooning of the left ventricle; **(B)** Echo-PIV analysis, revealing the presence of a single vortex, occupying the center of the left ventricle and non-interacting with others (arrow). **(C,D)** PIV derived polar histograms showing a relatively preserved base-apex flow direction. Adapted with permissions from Cimino et al. (43). Particle image velocimetry (PIV), left ventricle (LV), left atrium (LA), aorta (Ao).

Echocardiographic particle imaging velocimetry (E-PIV) is an emerging technique for estimating intracardiac blood flow patterns (41, 42). To date, a single case report described E-PIV findings in TTS (Figure 2), in which a relatively preserved intra-ventricular pressure gradient and energy dissipation were observed when compared to usual findings in AMI patients (43). Since reduced energy dissipation is associated with poorer LV function (44), E-PIV findings in TTS needs to be clarified to ascertain whether the more physiologically vortex behavior is a common feature in this condition, and if this could have any relationship with outcomes.

#### Magnetic Resonance Imaging

**Figure 3:**
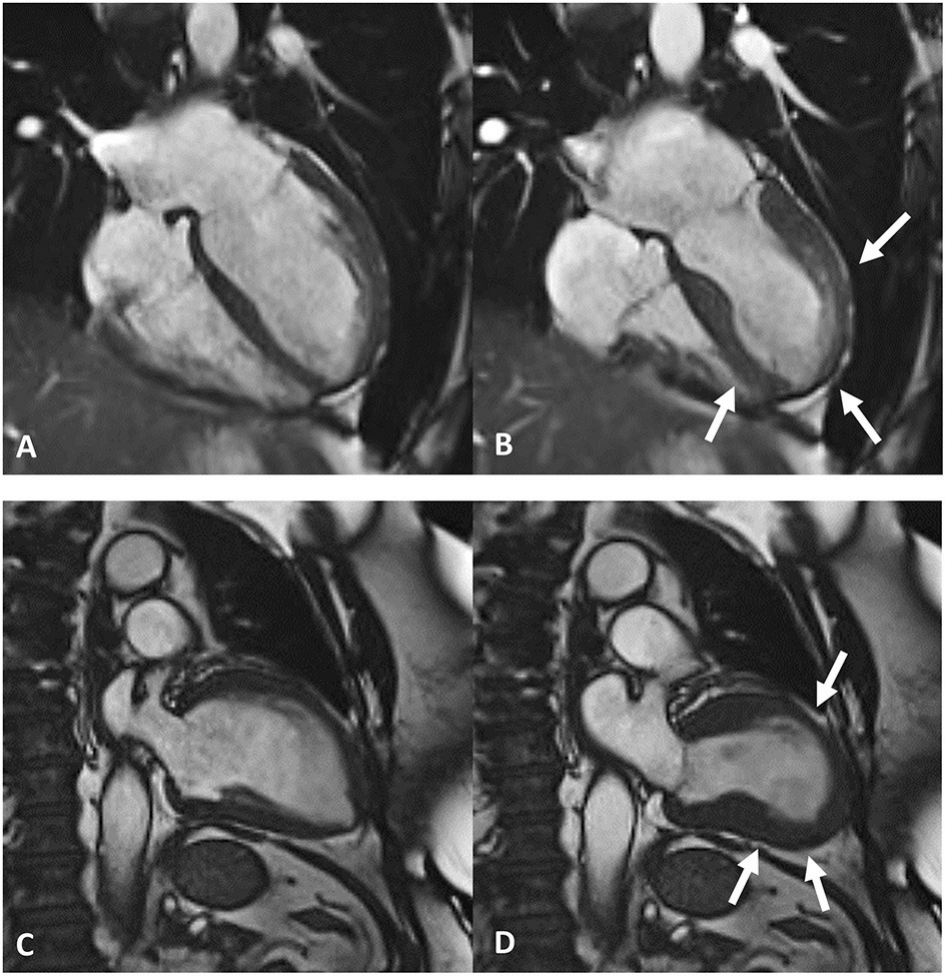
Frames taken from cardiac magnetic resonance cine imaging. Top row, end-diastolic **(A)** and end-systolic **(B)** frames depict apical ballooning of the left ventricles (white arrows in **B**). Bottom row, end-diastolic **(C)** and end-systolic **(D)** frames depict mid-ventricular ballooning of the left ventricles (white arrows in **D**).

Amongst cardiovascular imaging techniques, cardiac magnetic resonance (CMR) is the diagnostic gold standard for assessing cardiac volumes, function (Figure 3), and tissue characterization, allowing evaluation of edema, and replacement fibrosis (45).

Classic CMR appearance of TTS includes widespread myocardial edema in the absence of significant replacement fibrosis at late gadolinium enhancement (LGE) imaging, especially when strict criteria for identification are used (i.e., >5 standard deviation) (46). Notwithstanding this, variable rate of LGE detection have been described (47), and in some cases linked to worse prognosis (48). Hence, the presence of LGE should not be considered *per se* a CMR criteria to exclude TTS diagnosis, taking into account that bystander diseases conditioning the presence of replacement fibrosis might also be present (49), including coronary artery disease (50). Preforming a CMR examination is suggested in all suspected TTS patients (1), where its results might change the previously established diagnosis (51); however, taking into account the lower availability of CMR as compared to echocardiography, priority should be given to those atypical cases (such as patients who are males, with atypical ballooning patterns, suspected myocarditis or high cardiac troponin release) in which an alternative diagnosis is more likely (52). Focal TTS pattern represents an intriguing subset of atypical ballooning, in which segmental LV abnormalities more closely resemble those of AMI or myocarditis (30); in these cases, CMR imaging results (especially the presence of ischemic/non-ischemic LGE) must be carefully interpreted according to other clinical findings such as inflammatory biomarkers, red flags for myocarditis, occlusion of small coronary vessels, vasospasm, or coronary dissection. Notwithstanding, the boundary between TTS and other forms of MINOCA can sometimes be quite indistinct; in example, immune check-point inhibitor myocarditis might present with TTS-like transient myocardial dysfunction and absence of LGE at CMR imaging, making differential diagnosis a difficult task (53–56).

In summary, standard CMR techniques can be extremely useful to establish a TTS diagnosis against other forms of MINOCA, moreover, novel tools are emerging to grant quantitative analysis and more accurate prognostic information.

Feature-tracking CMR (FT-CMR) is a novel technique for evaluating cardiac chambers deformation in a similar fashion as ST-TTE does for echocardiography, though basic principles behind the two techniques are quite diverse (57). LV longitudinal, but not radial or circumferential, deformation analysis at FT-CMR aided differentiation between ballooning patterns and provided information regarding long-term outcome, outperforming evaluation of LVEF (58). Moreover, FT-CMR evaluation of LV rotational mechanics revealed transient dyssynchrony, more pronounced in the vulnerable subset of patients with stressful triggers, comorbidities and higher mortality risk (59). Acute phase left atrial functional impairment as assessed by FT-CMR is associated with long-term mortality, even when accounting for traditional cardiovascular risk factors and LVEF (60). This finding reiterates the view of TTS as causing global myocardium involvement, even beyond areas of visually assessed abnormal wall motion.

**Figure 4:**
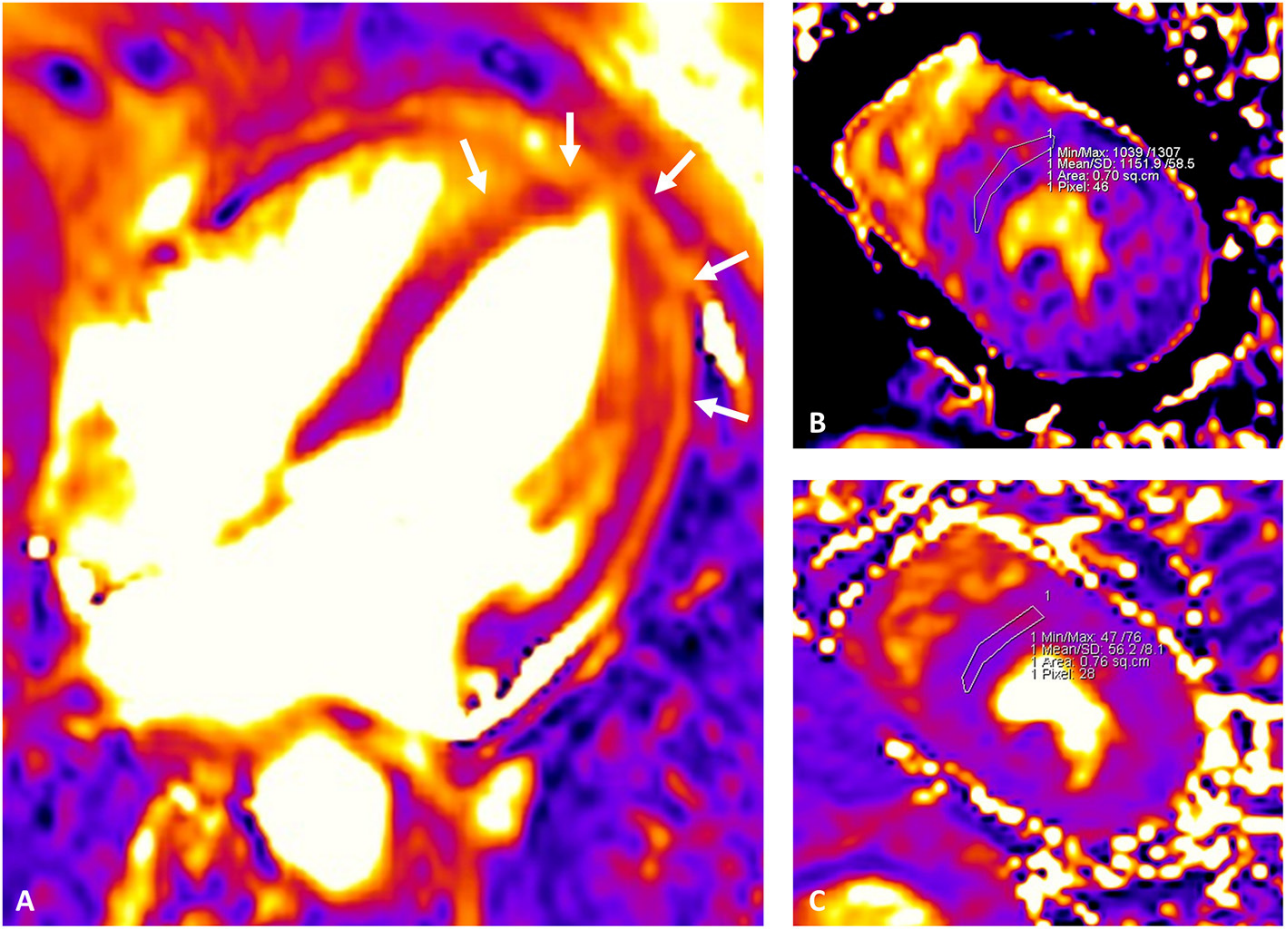
Cardiac magnetic resonance mapping images depicting myocardial edema in the acute phase of TTS. Mid-apical circumferential edema of the left ventricle is visualized as areas of higher intensity from the T2 mapping image (white arrows in **A**). Parametric quantification of native T1 and T2 values is better performed from the mid short-axis view by drawing a conservative region of interest (ROI) within the interventricular septum. Native T1 (MOLLI) **(B)** and T2 **(C)** shows a parallel increase at 1,152 and 56 ms, respectively. Extra-cellular volume (ECV) fraction was calculated from native and post-contrast T1 mapping images (mid-slice, short-axis, interventricular septum ROI) and hematocrit level as described in the literature (64), resulting elevated at 31%. The examination was performed with a 1.5 T scanner (Siemens Aera, Erlangen, Germany); in-center upper reference of normality for the sequence and vendor used are as follow: native T1 995 ms; native T2 49 ms, ECV 26%.

Recently developed CMR mapping sequences allow a parametric quantification of interstitial expansion in the myocardium, with signal intensity mainly depending on extra-cellular water (T2 mapping) as well as fibrosis and infiltration (native T1 and ECV). In TTS, marked increase of native T1 and T2 as well as ECV were observed in the acute phase (40, 61–63) (Figure 4). Interestingly, T2 shows direct correlation with native T1 and ECV (65), suggesting both a prominent role of extra-cellular myocardial edema in driving acute phase interstitial expansion (66), and a significant influence of myocardial edema on T1 mapping-derived measurements (both native T1 and ECV), as already demonstrated in multiple clinical settings (67, 68). Parametric edema quantification potentially retains prognostic value, since its presence and extent has been linked to both ECG abnormalities and potential TTS complications (69, 70). Higher T2 values within the first few days after the acute event were found in TTS patients with delayed recovery (62), as well as in those with lower LVEF at presentation. Notwithstanding, data on mortality are still lacking in this context, given the limited sample size of current CMR mapping studies in TTS.

In recovered TTS patients, native T1 has been described as persistently elevated when compared to that observed in a matched control group, even more than 1 year after the acute event (40). This finding was accompanied by impaired cardiac deformation (despite preserved LVEF), higher natriuretic peptide level and a persisting cardiac limitation observed on exercise testing at cardiopulmonary stress test, pointing toward subtle long-term non-transitory TTS related abnormalities. Moreover, magnetic resonance imaging including ultrasmall superparamagnetic particles of iron oxide enhancement showed signs of ongoing low-grade inflammation in the chronic phase (71). Further studies are needed to evaluate prognostic relevance of these persistent abnormalities.

#### Computed Tomography

Coronary computed tomography (CCT) is a non-invasive morphologic evaluation of the coronary tree, with an expanding role in the evaluation of patients with suspected coronary artery disease. In TTS presenting without ST-elevation at ECG, it can be reliably used in the acute phase to rule-out AMI (72, 73), also allowing a more accurate detection of coronary artery course abnormalities, such as myocardial bridging, quite common in TTS (73, 74). Some patients with TTS, especially those without an ST-elevation at presentation, with increased frailty and high comorbidity burden might benefit from CCT in order to avoid invasive procedures such as coronary angiography; in these cases, CCT can help confirming the diagnosis and providing a non-invasive assessment of bystander CAD that might be present even in TTS. Of note, this constitutes an important negative prognostic marker in TTS (50), whose identification can lead to significant changes in the therapeutic management such as more aggressive cholesterol treatment as well as long-term anti-aggregation.

When compared to matched control subjects, TTS patients were found to have increased peri-coronary fat attenuation index (pFAi), a measure associated with coronary artery inflammation (75). pFAi showed to be a risk factor for developing adverse cardiovascular events in the general population with suspected coronary artery disease (76). The easy evaluation of this measure from standard coronary computed tomography images makes it attractive to further investigate its potential association with outcome in TTS.

#### Nuclear Imaging

Single photon emission computed tomography (SPECT) with 201-thallium chloride has been used to investigate myocardial perfusion in TTS with conflicting results. Some studies reported a mild reduction of perfusion limited to the dis/akinetic segments in the acute phase, while others reported normal perfusion (77–80). Cardiac nuclear imaging was mostly used to investigate cardiac adrenergic function by SPECT with meta-iodobenzylguanidine (MIBG); a severe and persistent uptake defect was demonstrated in TTS patients, despite rapid normalization of myocardial perfusion, which suggested persistence of myocardial sympathetic dysfunction (81–84). Additionally, nuclear imaging may also investigate myocardial metabolism, and both SPECT using 123-I-β-methyl-iodophenyl pentadecanoic acid (which reflects fatty-acid metabolism) and positron emission tomography (PET) using 18-F-flourodeoxyglucose (which reflect glucose utilization) have shown persistently reduced metabolic activity in the impaired regions in TTS (80, 85–88).

However, up today, almost all nuclear imaging studies have been performed mainly for research purposes, while prognostic data are scarce. A recent study in a cohort of 90 TTS patients demonstrated that an extensive defect of 123-I-MIBG scintigraphy uptake appears to be associated with a higher rate of in-hospital complications (89). Patients with delayed improvement in LV function had significantly higher levels of catecholamine, higher washout rate in 123-I-MIBG and higher in-hospital complications. Consistently, it has been hypothesized that hyperactivation of autonomic nervous system and higher levels of norepinephrine may induce acute LV outflow obstruction and increased ventricular afterload, as well as elicit ventricular arrhythmias and subsequent sudden cardiac death (90, 91). Increased autonomic activity could also lead to delayed LV function recovery, which may be associated with complications due to heart failure (89). Hence, a severe defect of 123-I-MIBG scintigraphy uptake during the acute phase of TTS may identify patients at higher risk for in-hospital complications, while slower recovery of 123-I-MIBG uptake may identify those at a higher risk for TTS recurrence or for a worse long-term outcome.

#### Brain-Heart Axis

Both nuclear and non-nuclear imaging modalities have been used to investigate the brain-heart axis in TTS. The precise pathophysiological mechanisms of TTS are incompletely understood but there is considerable evidence that sympathetic stimulation is central to its pathogenesis (3), thus an underlying link between the brain and heart has long been proposed. Specifically, stress can activate the sympathetic nervous system and lead to a complex pathophysiologic cascade, including catecholamine toxicity, abnormal myocardial perfusion, myocardial stunning, and endothelial dysfunction (3).

The response to stressors is governed by an ensemble of neural structures, such as the limbic system (the amygdala, the cingulate gyrus, the hippocampus, the insula, etc.), the ventromedial prefrontal cortex and the brainstem. Recently, increasing interest has been directed toward the involvement of the brain–heart axis in in the pathophysiology of TTS. In 2014, Suzuki et al. first documented brain activation in three patients with TTS examining cerebral blood flow with single photon-emission computed tomography (SPECT). In the acute phase, the researchers observed a marked increase in brain activity in areas linked to abnormal stress-induced sympathetic arousal (brainstem, hippocampus, and basal ganglia). Furthermore, brain activation remained to some extent in the chronic phase, after full recovery of cardiac wall motion (92).

Later functional magnetic resonance imaging studies demonstrated structural alterations in the limbic networks of TTS patients during the acute (93) and chronic phases (94), while increased connectivity, in a network that included the left amygdala and the right insula, was shown after exposing patients with previous TTS to a local stress (cold) (95). Reduced functional connectivity in the limbic systems of a population of 15 female patients with previous TTS compared to healthy age- and gender-matched controls (96).

All of these studies investigated brain alterations in TTS patients after the acute event.

The study by Radfar et al. is the first to assess cerebral activity prior to the onset of TTS. The amygdala activity using [18F] fluorodeoxyglucose positron emission tomography/computed tomography (18F-FDGPET/CT) was measured retrospectively in 104 patients who underwent clinical 18F-FDG-PET/CT imaging, including 41 who subsequently developed TTS and 63 matched controls. Patients with subsequent TTS had higher baseline amygdala activity and, among the patients who developed TTS, those with higher amygdala activity developed TTS about 2 years earlier compared with those with lower level of amygdala activity (97).

These neuroimaging findings demonstrate structural and functional alterations of stress-related brain networks in patients with TTS even before the acute event, suggesting long-lasting psychological stress. Chronically heightened stress-associated neural activity may hypothetically induce an individual to react to subsequent stressors with a more vigorous neurophysiological response, thus increasing TTS risk (97). Consequently “heart-brain axis” could represent a potential target to reduce TTS risk.

New neuroimaging techniques could be useful in identifying patients at high risk of TTS recurrence, suggesting a longer follow-up and the implementation of both pharmacological and non-pharmacological behavioral therapy (i.e., stress reduction).

However, additional randomized prospective trials and new interdisciplinary approaches are required to further investigate the role of the brain-heart axis in the pathogenesis and prognosis of TTS.

### Genetic Markers

Table 3 summarizes the main results from studies on TTS genetic markers. Both familial (98, 99) and recurrent cases (24, 100) of TTS have been described, suggesting a possible influence of the genetic background in the pathogenesis of the syndrome. Single nucleotide polymorphisms (SNPs) belonging to both adrenergic pathway and estrogen receptors genes have been related to higher predisposition for developing TTS. One study reported the association between the Arg389Gly substitution within the adrenergic receptor B1 and TTS occurrence (101). However, these data were not confirmed by other reports (102–104). On the contrary, the Gln27Glu substitution within the adrenergic receptor B2 was observed more frequently in healthy controls than in TTS patients (101). SNPs linked to TTS involve the regulatory function of the anti-apoptotic protein Bcl-2-associated athanogene 3 gene, which likely contributes to myocyte stress resistance (105), and the rs2234693 within the estrogen receptor 1 gene which has been associated with higher risk of TTS occurrence (106). However, all mentioned studies are limited by a gene-target approach and an incomplete analysis of the whole adrenergic system pathway. Only a single study performed a whole-exome sequencing for genes related to catecholamines and adrenergic signaling in 28 TTS patients, and revealed no difference in the allelic frequencies between TTS patients and controls (107).

**Table 3:** Table summarizing studies on TTS genetic markers.

**Protein name**	**SNP**	**Protein variation**	**Effect**	**Clinical outcomes**
β1 adrenergic receptor	rs1801253	Arg389Gly	Gain of function	More frequently found in TTS patients No significant difference between TTS and healthy controls
β2 adrenergic receptor	rs1042714	Gln27Glu	Resistance to downregulation	More frequently found in healthy controls
Bcl2-associated athanogene 3 (BAG3)	rs8946	3' UTR	Altered cellular response to epinephrine	More frequently found in TTS patients
Estrogen receptor 1	rs2234693	–	unknown	Higher risk of TTS development in carriers

A relatively larger genome-wide association study reported findings from 96 TTS patients (108). Several promising candidate loci were identified, mostly linked to traits as psychiatric disorders, blood pressure, thyroid disease and cancer, further highlighting the role of comorbidities in the genesis of TTS (109).

In summary, current data on the genetic features of TTS only suggest a genetic etiology of this pathological condition. However, we are becoming aware that, whereas environmental triggers and concomitant comorbidities are pivotal in TTS development, the genetic heterogeneity and a potential polygenic predisposition may also play a contributory role mainly by determining the dysregulation of the adrenergic system. Larger cohorts are required for a better evaluation of the impact of genetic background on TTS occurrence and prognosis.

### Clinical Perspective

Despite similar long-term outcomes, the nature of adverse events in AMI and TTS is different. Indeed, TTS is featured by mainly non-cardiovascular mortality (19, 21, 110–112), even during the acute phase characterized by different degrees of LV dysfunction (11). However, cardiovascular assessment still provides fundamental clinical and prognostic information in TTS, both in the short and in the long-term. Indeed, a higher cardiac involvement as detected by imaging modalities or cardiac biomarkers [especially natriuretic peptides, (113)] is associated with worse prognosis, even after recovery of left ventricular ejection fraction. In this view, the prognostic power of cardiac imaging in TTS should be interpreted as the ability of identifying both the cardiovascular consequences of TTS and those underlying pre-existing characteristics of vulnerable phenotypes prone to heart failure. In this perspective, broader acute cardiac dysfunction or long-term abnormalities might just be a proxy of a wider comorbid state, which is actually the condition driving prognosis (114, 115). Advanced cardiac imaging might still provide reliable prognostic information (116) and should be considered, if available, in every patients with previous TTS even though appropriate therapies to improve outcome in these patients remain to be identified. Gaps in knowledge remain as to whether patients with previous TTS, recovered left ventricular ejection fraction, and persisting subtle cardiac abnormalities might benefit from specific therapies. Observational data indicate a lower long-term mortality in TTS patients treated with angiotensin-converting enzyme inhibitors (10). To this extent, a prospective ongoing trial is currently investigating the effect of N-Acetylcysteine and ramipril on edema resolution at CMR and longitudinal strain improvement in patients with acute TTS (117).

Genetic investigations, rather than cardiac imaging, may play an important role in risk stratification in the particular setting of the predisposition to TTS recurrence. However, where clinical implementation of genetic testing implies a multidisciplinary approach with genetic counseling and ethical considerations (118), currently available evidence still limits its applicability in the clinical field.

## Conclusions

Patients with TTS may benefit from advanced cardiovascular imaging tools offering unique information to assist in short- and mid-term risk stratification, well beyond traditional assessment of left ventricular ejection fraction. Due to the limited robust evidence, genetic evaluation does not currently provide significant advantages in guiding the clinical management. Hopefully, future studies aimed at better characterizing the genetic background of TTS may identify useful markers that could assist in predicting disease recurrences and help in familial screening.

## Author Contributions

LA and LL designed the study. LA, LL, CA, MS, and GT drafted the manuscript. All authors provided relevant comments, revised the draft, and approved the final version of the manuscript.

## Conflict of Interest

The authors declare that the research was conducted in the absence of any commercial or financial relationships that could be construed as a potential conflict of interest.

## Publisher's Note

All claims expressed in this article are solely those of the authors and do not necessarily represent those of their affiliated organizations, or those of the publisher, the editors and the reviewers. Any product that may be evaluated in this article, or claim that may be made by its manufacturer, is not guaranteed or endorsed by the publisher.
